# Supplementary figures and images for: Human decision making balances reward maximization and policy compression
Source: PLoS Comput Biol. 2024 Apr 26;20(4):e1012057. doi: 10.1371/journal.pcbi.1012057 (PMC11078408; doi:10.1371/journal.pcbi.1012057)

# Policy compression models

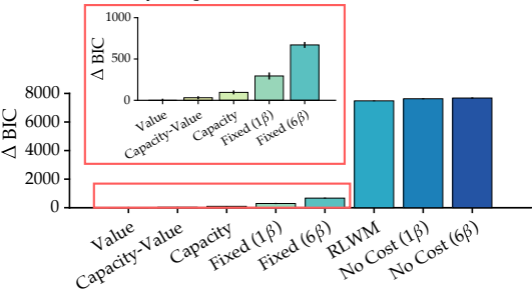

Supplement: S1 Fig — The difference in Bayesian Information Criterion (BIC) relative to the model with the lowest BIC (Value). (Inset) A zoomed-in view of all the policy compression models. Policy compression models in general outperform the RLWM and Standard RL models. Error bars indicate standard error. (PDF) [file pcbi.1012057.s001.pdf]

A

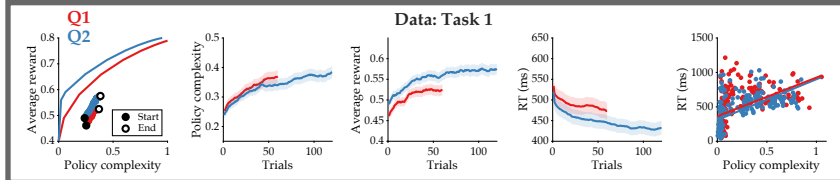

B

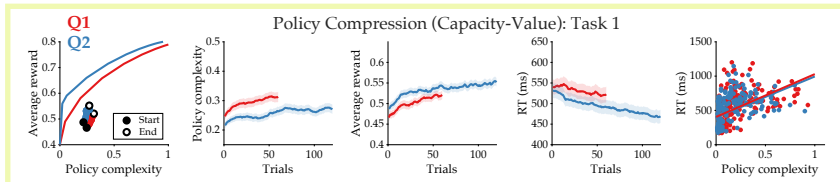

C

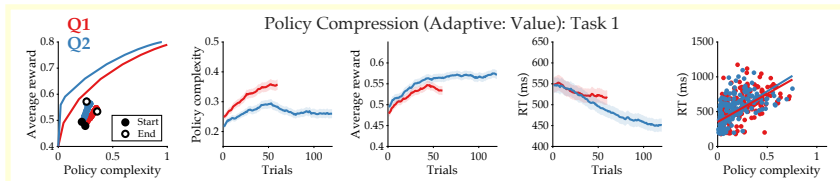

D

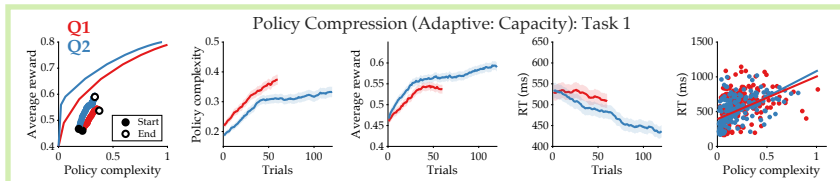

E

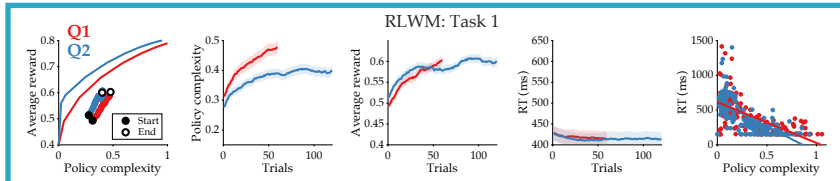

F

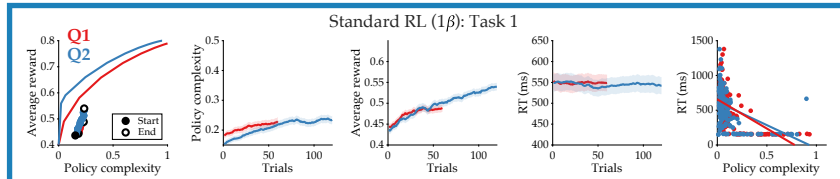

Supplement: S2 Fig — (A) From left to right: The dynamic reward complexity trade-off, averaged across all subjects. Solid dot indicates the start, while open dot indicates the end, of learning. Policy complexity, average reward, and response time (RT) as a function of trials. Response time as a function of policy complexity. Note that policy complexity, average reward, and RT are computed via a sliding window of 30 trials. The running average in each plot is therefore truncated to 30 trials fewer than the total number of trials, as there are not enough elements to fill the window at endpoints. (B) Same as (A) but data simulated from the winning policy compression model (Adaptive: Capacity-Value). (C) Same as (A) but data simulated from the Adaptive: Value model. (D) Same as (A) but data simulated from the Adaptive: Capacity model. (E) Same as (A) but data simulated from the RLWM model. (F) Same as (A) but data simulated from the No Cost (1β) model. All shaded error bars indicate standard error. (PDF) [file pcbi.1012057.s002.pdf]

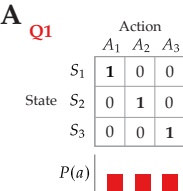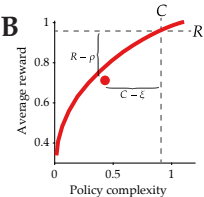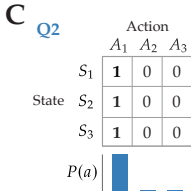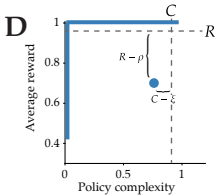

Supplement: S5 Fig — Two task conditions illustrating how the reward function affects the distribution of actions and changes the reward-complexity trade-off. (A) In this example condition, there is one unique rewarded action for each state. (B) This results in a roughly uniform marginal action distribution and a strictly monotonic reward-complexity trade-off. (C) In this condition, all states share the same rewarded action, causing the marginal action distribution to be heavily biased towards one action. (D) This reward structure results in a non-strictly monotonic reward-complexity trade-off. Note that in this condition, agents could achieve the highest average reward value with a variety of policy complexities. The example points on each plot show different suboptimal policies that can move in the reward-complexity space depending on how β is being updated using the capacity limit C, aspiration level R, or a combination of both. (PDF) [file pcbi.1012057.s005.pdf]
