# Supplementary material for: Human decision making balances reward maximization and policy compression
Source: PLoS Comput Biol. 2024 Apr 26;20(4):e1012057. doi: 10.1371/journal.pcbi.1012057 (PMC11078408; doi:10.1371/journal.pcbi.1012057)
Supplement: S3 Fig — (A) From left to right: The dynamic reward complexity trade-off, averaged across all subjects. Solid dot indicates the start, while open dot indicates the end of learning. Policy complexity, average reward, and response time (RT) as a function of trials. Response time as a function of policy complexity. Note that policy complexity, average reward, and RT are computed via a sliding window of 30 trials. The running average in each plot is therefore truncated to 30 trials less than the total number of trials, as there are not enough elements to fill the window at endpoints. In the data, the policy complexity is similar across conditions and increases slightly over the course of the task. While the Capacity-Value model (B) captures this between-condition similarity, the Value (C) and Capacity (D) models predict diverging policy complexities. (B) Data simulated from the winning policy compression model (Adaptive: Capacity-Value). (C) Data simulated from the Adaptive: Value model. (D) Data simulated from the Adaptive: Capacity model. (E) Data simulated from the RLWM model. (F) Data simulated from the No Cost (1β) model. All shaded error bars indicate standard error. (PDF) [file pcbi.1012057.s003.pdf]

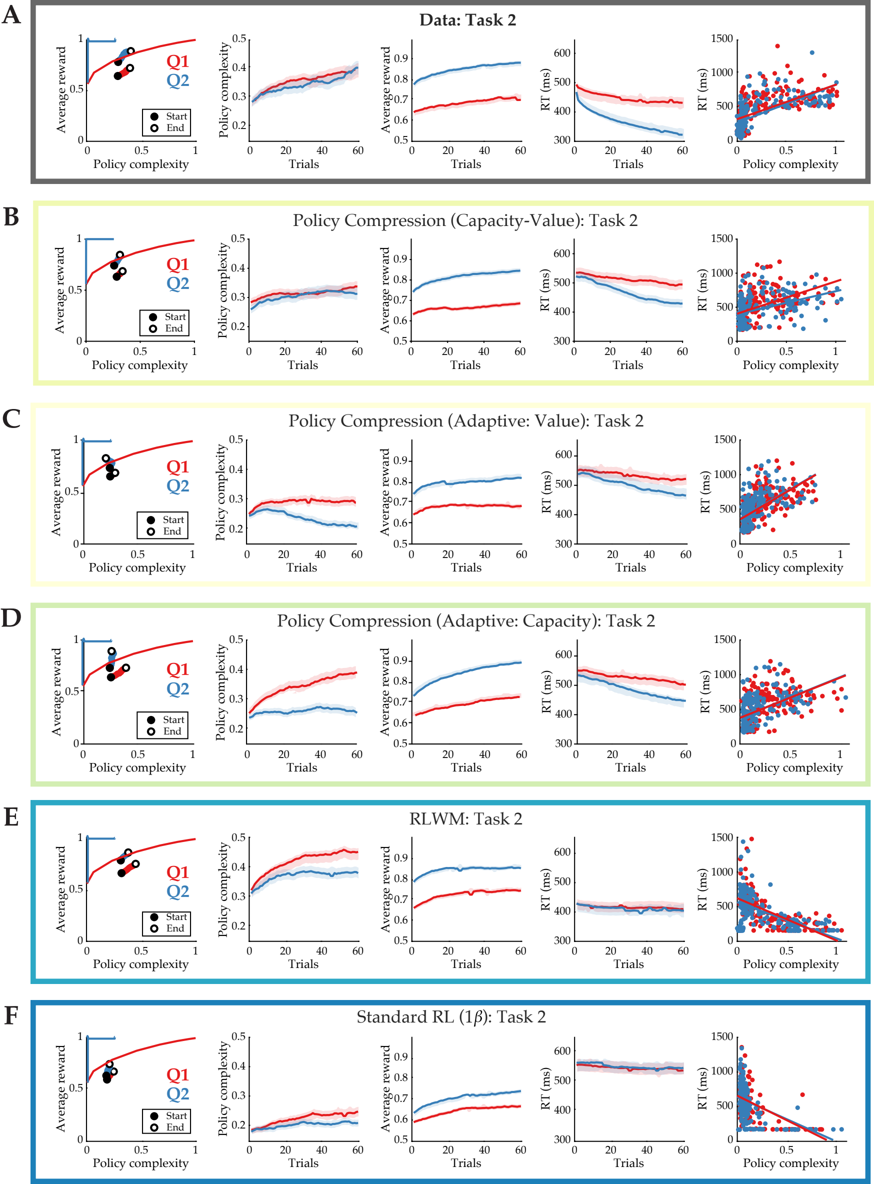

A
Data: Task 2
B
Policy Compression (Capacity-Value): Task 2
C
Policy Compression (Adaptive: Value): Task 2
D
Policy Compression (Adaptive: Capacity): Task 2
E
RLWM: Task 2
F
Standard RL (1β): Task 2
Average reward
Policy complexity
Q1
Q2
Start
End
Trials
RT (ms)
