# Supplementary material for: Human decision making balances reward maximization and policy compression
Source: PLoS Comput Biol. 2024 Apr 26;20(4):e1012057. doi: 10.1371/journal.pcbi.1012057 (PMC11078408; doi:10.1371/journal.pcbi.1012057)
Supplement: S4 Fig — (A) From left to right: The dynamic reward complexity trade-off, averaged across all subjects. Solid dot indicates the start, while open dot indicates the end of learning. Policy complexity, average reward, and response time (RT) as a function of trials. Response time as a function of policy complexity. Note that policy complexity, average reward, and RT are computed via a sliding window of 30 trials. The running average in each plot is therefore truncated to 30 trials less than the total number of trials, as there are not enough elements to fill the window at endpoints. In the data, policy complexity remains roughly constant in both conditions, though it is overall higher in Q1 than in Q2. This trend is successfully mirrored by the Capacity-Value model (B). In the Value (C) model, the complexity difference between conditions is much smaller, while in the Capacity (D) model, complexity starts at the same point for both conditions and diverges with learning. Additionally, average reward steadily increases for both conditions, though it is always higher on average in Q1 than Q2. This overall difference in reward, although less pronounced, is captured by the Capacity-Value model but not by the Capacity and Value only models. (B) Data simulated from the winning policy compression model (Adaptive: Capacity-Value). (C) Data simulated from the Adaptive: Value model. (D) Data simulated from the Adaptive: Capacity model. (E) Data simulated from the RLWM model. (F) Data simulated from the No Cost (1β) model. All shaded error bars indicate standard error. (PDF) [file pcbi.1012057.s004.pdf]

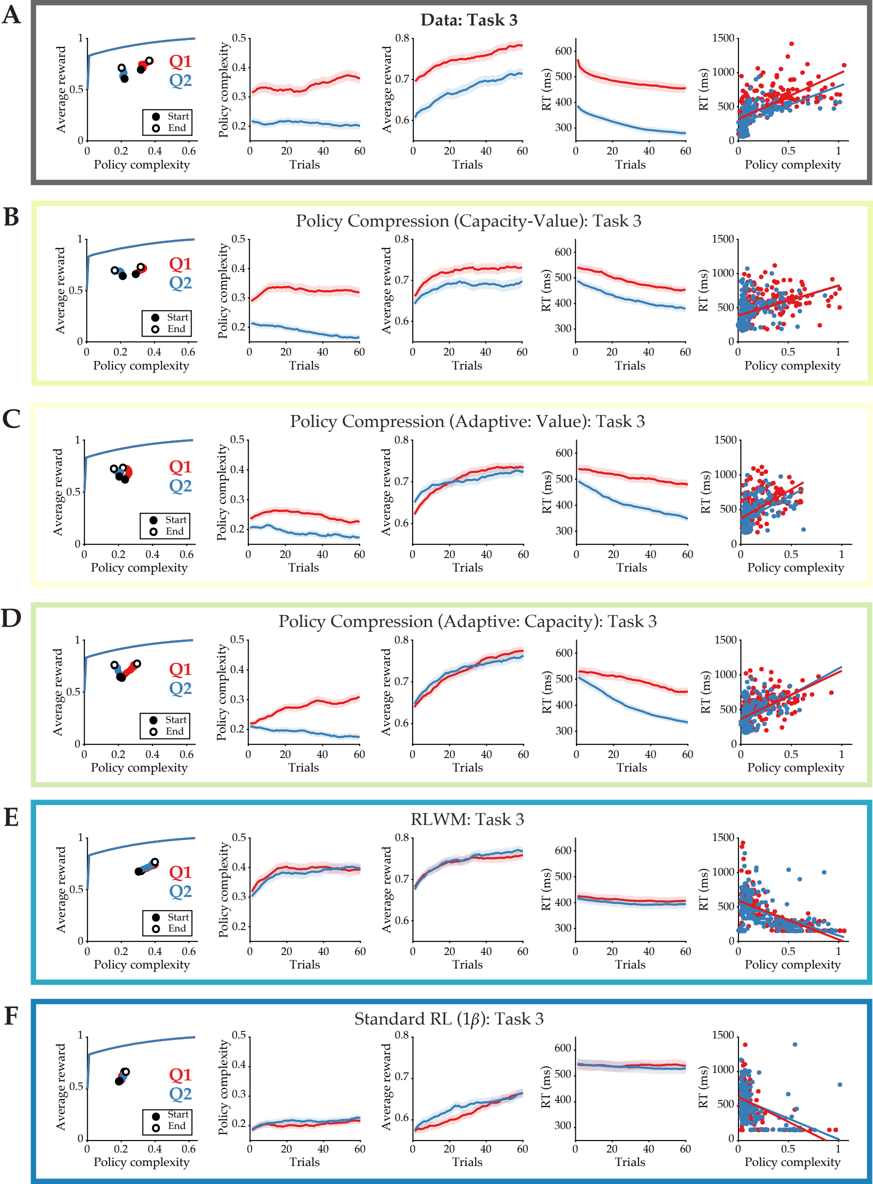
A
Data: Task 3
B
Policy Compression (Capacity-Value): Task 3
C
Policy Compression (Adaptive: Value): Task 3
D
Policy Compression (Adaptive: Capacity): Task 3
E
RLWM: Task 3
F
Standard RL (1β): Task 3
Average reward
Policy complexity
Q1
Q2
Start
End
Trials
RT (ms)
