# Supplementary material for: Human decision making balances reward maximization and policy compression
Source: PLoS Comput Biol. 2024 Apr 26;20(4):e1012057. doi: 10.1371/journal.pcbi.1012057 (PMC11078408; doi:10.1371/journal.pcbi.1012057)
Supplement: Attachment — Submitted filename: lai_gershman_response_to_reviewers.pdf [file pcbi.1012057.s006.pdf]

Dear Dr. Beierholm & Dr. Serre,

Thank you for giving us the opportunity to revise our manuscript in response to the reviewer comments, which were extremely helpful. We respond to each comment point-by-point in green below. We have included the new manuscript as well as a copy that indicates all tracked changes. Here we summarize the major changes made:

- We restructured the Results section by moving most of the involved details of the model to the Methods and rewriting a simpler overview of the theory that helps readers grasp its main intuitions as they relate to the behavioral experiments that follow. We hope this allows the spotlight to be on the experimental predictions and helps readers quickly grasp the implications of theory.
- We significantly changed Figure 2 as a visual aid to the condensed modeling section in the Results.
- We significantly changed Figure 8 to explore the relationship between action bias and policy complexity in a more detailed way.
- We added one Figure 9 showing how policy complexity is correlated across tasks.

Thank you for your consideration, and we look forward to hearing back from you again!

Sincerely,
Lucy Lai and Samuel Gershman

Reviewer #1

In their manuscript, 'Human Decision Making Balances Reward Maximization and Policy Compression,' the authors explore decision-making and learning processes, and their underlying cognition in humans, by developing a new policy-compression theory. According to the theory, in a reinforcement-learning (RL) context, the mapping between states and actions, the policy, is characterized by complexity, the extent to which action selection is state-specific vs. shared across states. Considering the limited capacity of human decision-makers' policy complexity, the authors use information-theory formalism to test several hypotheses regarding the expected reward, stochasticity, and reaction time that would be realized under different conditions. In three novel experimental tasks with a within-subject design, the authors modify the distribution of stimuli (states) presentation frequency to be more or less uniform (task 1), the extent to which optimal actions are shared across states (task 2), and the time allotted for decision making (task 3). Across these tasks, and using previously published data, the authors find support to key predictions of the policy-compression theory. The policy compression theory fares well in quantitative model-comparison measures, as well as replicates qualitative behavioral patterns. Taken together, this evidence suggests that the policy compression model goes above and beyond the predictions of standard reinforcement learning (RL) models, as well as RL models limited by a working memory capacity (RLWM).

I really enjoyed reading this well-written manuscript. The elegant development of the policy-complexity theory and its application to human cognition makes an important contribution, offering a consolidated framework for theories from different domains, including bounded rationality and reinforcement learning. By doing so, the authors substantially promote a novel understandings of human cognition.

My one main comment is on the focus and the organization of the manuscript. While I believe that addressing this comment may assist future readers, this is largely a matter of style. So, I only offer this as a suggestion and leave it to the authors' discretion.

In its current form, the manuscript gives similar weight to the presentation of the policy compression theory,

which was partially developed in previous publications, and the novel experiments with their respective results. I highly appreciated the comprehensive presentation of the theory, allowing this manuscript consistent notation and having it as a self-contained unit. However, for me, a greater focus on the experiments and their results would have made the reading clearer while motivating the reader to seek a deeper understanding of the theory. Specifically, the Introduction does a great job of presenting relevant background but uses a lot of terminology that the naive reader may not be familiar with. Such presentation requires the readers to invest in familiarizing themselves with substantial new terminology, without yet knowing which parts of this theory would be relevant and why. Similarly, sections 2.1-2.4 present, very clearly, the policy-compression theory, but the reader does not know yet to what end. An alternative approach could be to use the Introduction for a more intuitive presentation of the basic concepts of policy compression, and how these may be realized under the different conditions tested in tasks 1-3. Such focus on hypotheses may allow the general reader a fluent understanding of the main concepts. Restructuring the Results section to present the experiments and their results upfront would allow general readers to quickly grasp the practical implications and utility of the theory, without the immediate need to understand the formal aspect of the theory, while still offering interested readers the in-depth theoretical exposition in subsequent sections.

Thank you. This was a very helpful suggestion and we 100% agreed with it! We tightened up the Introduction as well as the beginning of the Results. We created a new Figure 2 to help people develop intuition for how the policy compression model makes unique predictions from a standard RL model. We hope this allows the spotlight to be on the straightforward experimental predictions and helps readers to quickly grasp the practical implications of theory.

Minor comments:

1. Complexity as a feature of participants (e.g., "individuals with low complexity") is mentioned throughout the text but I was not sure exactly what does that mean – is it the per-block policy complexity inferred from behavior?

Yes, it's the per block empirical policy complexity estimated via the mutual information between the states (stimuli) that participants saw on each trial, and their corresponding actions. We have added some information in line 396-399 to make this clearer: "Furthermore, we reasoned that subjects with low complexity (defined as the empirical policy complexity estimated from choice behavior in each task condition) would benefit more from action bias, since it is more efficient to focus one's limited cognitive resources on states (and their optimal actions) that appear most frequently."

2. In task 3 the authors test the effects of time pressure on policy compression. I wondered whether the model could provide some quantitative insights into the cognitive mechanisms involved in the hypothesized compression. Namely, **whether it is possible within the framework of the specific model used to estimate the improvement in compression per unit of time.**

This depends on the slope of the linear relationship between RT and policy complexity. Empirically speaking, this varies depending on the study and the imposed time constraints for responding. Generally, we've found higher slopes (bigger increase in RT per unit complexity) when RT is unconstrained. When RT is constrained to $0<RT<2$ secs (as in our experiment), the slope seems to be on average around 600ms. So 1 bit of information takes ~600ms to execute. The definitive way to answer this question would be to run another experiment where we only applied time pressure after learning. This would be a potential follow-up experiment. However, given that it's somewhat tangential to the main thrust of our paper, we chose not to pursue this follow-up.

3. In the following sentence and throughout the text, the manuscript interprets reduced complexity under time pressure as a feature of time-dependent decoding: "… individuals reduce their policy complexity under time pressure, providing evidence for the hypothesis that actions are selected through time-sensitive decoding of a

compressed code". However, if I understood correctly, in the presented tasks decision-making was intertwined with learning. So, a priori, time pressure may affect the learning part as well (policy formation and its encoding). I wondered if there was a reason to focus the interpretation on the decoding part.

This is a good point, and we've addressed the nuance in lines 488-494: "We note one caveat that in this study we did not explicitly disentangle policy learning from its implementation, which raises the question of whether reduced complexity under time pressure is a feature of time-dependent *decoding* alone, or whether it a priori affects policy *encoding* as well. However, since previous work [Lai & Gershman, 2021; Filipowicz et al., 2020] has shown that policy compressibility relates to its learnability (i.e., simpler or more compressed policies are easier to learn), it is reasonable to assume that time pressure also causes agents to learn overall simpler policies."

4. The manuscript outlines a spectrum of policies varying from policies with state-specific actions (higher complexity) to policies with actions that shared across states (lower complexity), which appears to align with the distinction between **model-based and model-free reinforcement learning**. A discussion on the intersection between these theories and potential implications may provide a broader context for interpreting the results.

We believe that these are orthogonal notions of complexity, as discussed by Filipowicz et. al. (2020) and Gershman (2020). To clarify this, we added the following in lines 533-544 into the Discussion: "Our study also highlights an important distinction between policy complexity (the number of bits needed to encode and decode a policy), statistical complexity (the amount of data needed to learn a policy), and computational complexity (the number of operations needed to execute an algorithm), which is important for placing our model in the broader context of other common RL algorithms such as model-free and model-based RL. For example, while model-based RL is more computationally complex than model-free RL (as it requires more operations to implement), it is not necessarily more statistically complex [Filipowicz et al., 2020], and also does not necessarily require a more complex policy. Since model-free and model-based RL both require attention to the state for selecting actions, the complexity of the policies learned with either algorithm will vary depending on the particular state space and reward structure that learning takes place in. A more comprehensive theoretical analysis of how policy complexity differs in common RL algorithms would be an interesting area for further exploration."

5. **Minor errors in the text:**
a. Figure 2: the caption might be confusing left and right.

Thank you for catching this! We corrected it.

b. The following seem grammatically incorrect:
i. "This parameter … assumes that time pressure would compress agents' policies via a decrease their capacity limit C or aspiration level R for that condition."

Changed in line 754 to: "This parameter … modeled the effect of time pressure as a reduction in the agent's capacity limit (C) or aspiration level (R), forcing the agent to further compress their policies."

ii. "we hypothesized that time pressure would compress agents' policies via a decrease their capacity limit C for that condition"

Changed in line 761 to: "We hypothesized that time pressure would further compress agents' policies by decreasing their capacity limit (C)"

Reviewer #2

This study describes a set of experiments testing a computational model of learning under cognitive computational constraints, called “policy compression”. This model views learning as a rate-distortion problem of finding the maximal obtainable Value of a learned policy under a constraint on its Complexity. As in standard reinforcement learning models, the policy is defined as a stochastic mapping from states to actions and its Value is the expected accumulated reward. Building on an information-theoretic learning framework previously developed by Tishby and others, the authors define a policy's Complexity as the mutual-information between states and actions, i.e., how much information, on average, does an agent’s selected action contain about the underlying state eliciting it.

The main findings are that human choice behavior and reaction times in instrumental learning tasks are better explained by the policy-compression model compared to other reinforcement learning based models. Specifically, people are able to utilize statistical redundancy in their environment (state, action and reward distributions) to compress their policies, i.e., reduce their complexity) resulting in preservative behavior that is biased towards frequently selected actions. They also found that policy complexity is reduced under time pressure, based on the hypothesis that action-selection requires a time-dependent decoding of the compressed state representation.

Overall, this is an elegant contribution to the growing literature demonstrating the usefulness of information-theoretic principles for elucidating the mechanisms and trade-offs underlying learning and cognition. My main concerns and more substantial comments are detailed under “General comments” below, whereas smaller issues and corrections appear “Minor points”.

General comments:

The issue of learning dynamics, namely how does the value-complexity tradeoff evolve throughout learning, deserves a more detailed treatment in the main-text, rather than being relegated to the supplementary (Figs. S2-S4). For example, in several occasions the authors mention “individuals with low complexity” (lines 78, 557) but it is likely that individual’s complexity evolves throughout the learning process and it would be interesting to see, for example, whether different subjects exhibit different learning dynamics, i.e., different beta update profiles, throughout learning. In particular, previous studies have reported a non-monotonic evolution profile of the policy complexity, in both biological and artificial learning settings, with an emphasis on value optimization at the early stage of learning shifting to compression at a later stage. A similar trend may be seen, at least in tasks 1 and 2, for the Policy Compression (Adaptive: Value) model (Figs. S2-S3) but not for the other models or data (or in task 3). It would be interesting if the authors could comment on this effect and why it appears only in this model variant.

We agree that the dynamics of learning are very interesting, but the reason we relegated it to the supplemental material is because our data in the end were not quantitatively diagnostic of any particular update model. While we concluded that the Capacity-Value model has the closest match to data, this is mostly a qualitative claim, as the quantitative model comparison bore mixed results. We emphasize that the main goal of the paper was to test the overarching behavioral signatures of the policy compression theory (in earned reward, complexity, stochasticity, and RT), which vary as a function of the state, action, and reward distributions. Furthermore, on any random sample of the subjects, the simulated evolution of complexity and value is not always consistent. We feel that the detailed analysis of learning dynamics could be an interesting topic for a future study.

Since the marginal action probability, P(a), depends on the policy (and vice-versa), it would be helpful to clarify with respect to which policies are the marginal distributions in Figs. 4-6 (subfigures (A)) computed. Are they derived from the optimal unbounded policy (with beta=infinity) or from a policy with a finite beta value estimated from the data?

We now clarify this in the figure captions for Figure 2A and Figure4A (and leave it implicit for the other similar figures): "The P(a) depicted for each task condition is derived from the optimal unbounded policy, which assumes that $\beta \to \infty$ (i.e., assuming that subjects perfectly learn the reward function)." An even simpler way to view it is simply the reward function probabilities averaged across states.

More importantly, this circular dependency between the policy and the marginal action distribution can lead to degeneracies in the definition of the optimal tradeoff. For example, there are multiple state-independent action distributions with zero complexity (delta function, uniform etc.). Does the theory make any specific predictions regarding such cases in which the "optimal" policy may be ill-defined?

As far as we know, our theory doesn't guarantee that there is a unique resource-rational policy. It does however guarantee that there exists at least one feasible policy on the optimal trade-off function.

Why do the authors compare their model with the RLWM model rather than other models which may seem more immediately related to the policy compression framework such as entropy-regularized RL or "empowerment" related frameworks? Alternatively, shouldn't beta_RL and beta_WM should be allowed to change (or at least be fitted) in analogy with the role of beta in the policy compression model? The authors should clarify the motivation for this choice and/or provide comparison with other, more immediately related models.

We chose RLWM (with RT) as our main competing model because it's a successful model of memory constraints in RL tasks, and because it was a natural comparison in terms of also giving concrete RT predictions (and how RT should relate to the policy), as we now note in line 181: "We chose the RLWM model as a natural point of comparison because it makes explicit how memory constraints affect performance and response time on RL tasks like the one we study."

We believe empowerment makes the complete opposite prediction, that people want to maximize policy complexity, and in any case is not really relevant to our study because it primarily has been used as an unsupervised pretraining objective, whereas subjects in our experiments received reward feedback. We have added a paragraph of the Discussion addressing empowerment (lines 522-528):

"A limitation of our theoretical framework is that it assumes people always prefer simpler policies (all other things being equal), whereas some evidence suggests a preference for *empowerment* (i.e., more complex policies) in certain situations, such as games without an explicit reward function [Brandle et al. 2023]. In such situations, policy complexity itself can become a source of intrinsic motivation. Intuitively, an agent with high policy complexity has more control over the environment, which is useful for carrying out many different tasks. An important challenge for future work will be to disentangle the conditions in which people seek or avoid policy complexity."

We address our rationale for fixing beta_RL and beta_WM in line 785-791 onwards: "Since beta_RL and beta_WM do not have the same interpretations as the beta in our model, we chose to fix them to the values stated in McDougle & Collins (it was their best fitting model), as the other parameters in their model were larger determinants of the model fit to data. Additionally, the RLWM model had a very large number of fitted parameters already (9) so adding even more would have contributed to potential degeneracy. We also note that changing the beta parameter would not have changed the qualitative predictions of the RLWM model."

The authors write (lines 96-98) that the theoretical framework of policy optimization under information-theoretic constraints was originally developed in their previous work (Lai, Gershman 2021, Gershman, Lai 2021). However, this attribution might be (unintentionally) misleading as such frameworks were developed at least a decade earlier, e.g. in (Tishby & Polani, 2010), (Parush, Tishby & Bergman 2011), that are indeed cited by the authors but in a different context. Also (Rubin, Shamir & Tishby 2012) develops a capacity constrained policy optimization framework in a more general setting of Markov Decision Processes. The authors should reference these earlier frameworks and elucidate in what ways they build upon or extend them (e.g. via the actor-critic process model or link to RT).

This is a reasonable criticism. We didn't mean to misattribute. We now clarify in lines 567-576: "The theoretical framework of policy optimization under an information-theoretic capacity limit was originally developed in several papers by Tishby and his collaborators [Tishby et al., 2011; Parush et al., 2011; Rubin et al., 2012]. Still and Precup [2012] and Lerch et al. [2018] developed online RL algorithms to learn a policy that optimizes the trade-off between reward and policy complexity through interactions with the environment. We further built upon these ideas [Lai et al., 2021; Gershman & Lai, 2021] by introducing a process model that (1) incrementally modifies the policy based on reward feedback that directly penalizes policy complexity, and (2) specifies how an agent's reward-complexity trade-off should evolve during learning. In the current study, we (3) built trial-by-trial response time predictions into our process model, which allows us to directly test the hypothesis that RT in part reflects the time-sensitive decoding of a compressed code. In what follows, we review the general framework along with the process model."

Eq. 8 and lines 129-131: shouldn't this relation hold for all points on the optimal reward-complexity trade-off curve (rather than just at the capacity constraint I(S;A)=C)?

Yes, but more precisely: each point on the optimal reward-complexity trade-off curve indicates the performance of an optimal policy at that particular capacity C. We hope it is now clearer in our revised text in lines 610-612: "In other words, at each value of C there exists a unique beta that constitutes one point on the optimal reward-complexity trade-off, and thus the entire trade-off curve (such as in Fig. 1C) is constructed by evaluating Eq. 10 at different values of C."

Eq. 10: the authors should clarify what assumptions (if any) are implied by choosing this specific parametric form for the class of admissible policies. Is it guaranteed to converge to the optimal solution of the variational problem (Eq. 6)?

We don't have a convergence guarantee for this algorithm, though it is based on regularized policy gradient algorithms that are widely used in machine learning. Our primary goal is not to develop convergent algorithms but rather to explain our empirical data.

Also, theta_(s,a) seems to be a proxy for Q(s,a). If so, why is it updated by the full policy cost (Eq. 11), including the complexity term rather than just the reward? Similarly, why is theta updated using an actor-critic model whereas P(a), xi and rho are updated via an exponential moving average. Finally, why are only the alpha_xi and alpha_rho learning parameters specified as fixed (at 0.01)?

These are good questions. The reviewer is correct that one could use Q(s,a) instead. We address this point on lines 644-651: "At this point, the reader may wonder why the policy parameters are updated according to the policy gradient rather than simply treated as state-action Q-values and updated directly based on reward, as suggested by the form of the optimal resource-constrained policy. While this might be more straightforward for small state and action spaces where we can use look-up tables, applications of this framework to

high-dimensional or continuous state and action spaces create challenges for value function approximators. In contrast, the optimal policy may be simpler to approximate with a relatively small number of parameters [Sutton & Barto, 2018]. Indeed, precisely because we are regularizing towards simpler policies, we expect this to be typically true."

As for the other parameters, we needed to pick an operationalization of our ideas that works in practice. You could replace the exponential moving average with some other kind of moving average, but we don't have the experimental resolution to make strong inferences about which is superior.

With regard to fixing some parameters, we did this to avoid degeneracy in the parameter space (since based on previous work, it is known that learning rates tend to trade off with one another). This is admittedly arbitrary, but again we are not making strong claims about these parameters in the first place. Our philosophy is that parameter identifiability is only really important if you want to make inferences about parameters, which is not the case for us.

Eq. 19: shouldn't beta on the r.h.s be replaced with 1? otherwise it is not clear to me how this results in the update dynamics described in the text below.

To be more specific about the update dynamics, We updated the text in lines 667-680 to the following to clarify: "This model variant adapts beta towards an approximation of the inverse slope (i.e., $(C-\xi)/(V-\rho)$) at a point on the optimal reward-complexity trade-off curve, and allows the agent to flexibly adapt to a variety of environments with different trade-off landscapes. For example, when the current policy complexity estimate ξ deviates from the capacity constraint C more than the current reward ρ deviates from the aspiration level R (i.e., the numerator is larger), beta should be updated towards a value greater than 1, and the policy should become more complex, or state-dependent (Fig 2, left). But when the deviation between current reward and aspiration level is greater than the deviation between policy complexity and capacity (i.e., the denominator is larger), the current policy is suboptimal and lies below the optimal trade-off curve (Fig 2, right). In this case, beta should be updated towards a value less than 1 to decrease complexity and move closer to the optimal reward- complexity trade-off. As a result, the agent may end up learning a policy that does not utilize its full capacity C, but that still optimally maximizes reward for the chosen policy complexity. In practice, we can not know what value of beta subjects use at the start of learning, so we initialize beta to a fitted value between 1 and 10 (see Methods for more details) and allow it to adapt via Eq. 25."

Fig. 4B: the policy complexity difference between the Q1 and Q2 conditions in the Policy compression model seem quite small, which perhaps explains why no significant difference in complexity was found in the data. Why didn't the experimental design include higher policy complexity contrast conditions?

We now clarify our points about compression in line 258: "Therefore, the signature of policy compression in Task 1 is being able to earn more reward in Q2 than in Q1 with the same policy complexity."

And in lines 288-292: "We note here that compression can occur in two main ways: (1) earning more reward with the same policy complexity (as seen in Task 1), and (2) learning a simpler policy while earning the same, or more, reward. Both are valid ways to "compress"' one's policy, as compression simply implies that people are ignoring some state information and taking advantage of the marginal action distribution."

We also note here that in our pilot studies of Task 1, we played with different state frequency manipulations, but in all the iterations, the effects were similar: participants took advantage of the task structure to earn **more reward** at the **same policy complexity** (strategy (1) as stated above).

Fig. 5B: how can the policy complexity level differ between Q1 and Q2 in the Standard RL & RLWM models (which are not supposed to be complexity sensitive)?

You are right that the standard RL & RLWM models are not supposed to be complexity sensitive, as there's nothing in the competing models that specifies what the policy complexity *should* be. However, it is possible to learn arbitrarily complex policies in the task and that is how we chose to simulate our comparison models. We now address this in lines 297-307 in the main text: "Note that for this task, the policy compression model makes similar qualitative predictions on most behavioral measures as the standard RL and RLWM models. While the Standard RL and RLWM models are not constrained by capacity limits, (i.e., there is nothing in the models specifying what the complexity of the optimal policy *should* be) they can still match subjects' empirical policy complexity through parameter setting (as we demonstrated through the examples in Fig 2B-D). So to enable a fair comparison, we simulated the comparison models to match the average policy complexity in our participant data. Though two different algorithms (e.g., Standard RL vs Policy Compression) can in theory generate the same policy complexity and reward, the pattern of their choice biases will differ significantly. This is exactly the case for Task 2, where the key behavioral signatures of policy compression are revealed when examining subjects' choice preferences in each condition (Fig 5C)."

Figure 8B: the blue and red lines seem almost overlapping but shouldn't there be overall lower complexity for the Q2 condition in task 2?

Sorry, this was actually a lack of clarity / confusing text on our part. The conditions depicted in the (old) figure were not Q1 and Q2, but rather different states from Q2. We have now completely changed the plot (see the new Fig 8).

Minor points:

Eq. 13: V is missing a caret.

Fixed, thank you.

Fig. 2 caption text: (Right) and (Left) should be switched (also, "monotonic" and "non-monotonic" should be "strictly monotonic" and "non-strictly monotonic" or something similar).

Fixed, and made the language more specific for monotonicity. Thanks for catching that mistake.

Line 206: missing the word "in" between "decrease" and "their".

Sentence was poorly worded, as pointed out by another reviewer, so we rewrote it entirely: "We hypothesized that time pressure would further compress agents' policies by decreasing their capacity limit (C)"

Line 210-1: parameter lists overflow page margins.

Fixed, thank you.

Eq. 26: how is phi defined?

Edited to the following: "…where phi represents time-based decay or forgetting of items held in short-term memory. Practically, phi is a fitted parameter that draws W (over all stimuli and actions) toward their initial values…"

Lines 268-272: specify what the parameters A, v_i,s_v,k_i, and d_i represent in the model.

We now elaborate on the meaning of these parameters in the paragraph from line 735-744: “To generate RTs in the No Cost and RLWM models, we draw each accumulator's starting point k_i from a uniform distribution on the interval [0, A]. By randomly-sampling the starting point from some range of initial bias (A), we create sources of extra random variability that allow for the initial amount of evidence for each action k_i to fluctuate from trial to trial. The drift rate of each accumulator d_i is then drawn from a normal distribution, N(v_i, s_v), where v_i is calculated from Eq. 38 using the respective pi for each model (i.e., Eq. 28 for the No Cost model and Eq. 33 for the RLWM model). The drift rate d_i represents the slope or speed of the accumulation process, which is proportional to the action probability specified by the current policy, according to Eq. 38. The variance of the drift rate distribution, s_v, also serves to inject random trial-to-trial variability in the evidence accumulation process for each action.”

Line 282: extra “e” at the end of “alternative”.

Fixed, thank you.

Line 289: explain what is meant by “probabilistic feedback”.

We now provide a bit more detail in lines 194-197: “After making a response, subjects were given feedback indicating whether their response was “correct"' (a reward of +1 is earned) or ``incorrect'' (no reward is earned). The probability that the subject would receive ``correct'' feedback given their response was defined by a reward function that varied across tasks and conditions.”

Line 336: Fig 5A should be 4A (and Fig 5B should be 5A in line 345).

Fixed, thank you.

Fig 5B - why is the axis range different for the Data Policy complexity plot?

That was a complete oversight! Thank you, we’ve fixed it for both Fig 5 and Fig 7.

Line 477: the word “was” appears twice.

Fixed, thank you.

Fig. 8: specify in the caption text what each point represents (trial/subject) and maybe add a legend to remind that Q1 is red and Q2 is blue (the colors also seem slightly different here compared to previous figures).

Added legend and fixed colors to be consistent with previous. Also changed the caption.

Table 1, line 6, “I” should be xi.

Fixed, thank you.

Fig. S2: why does the Q1 condition have less trials than the Q2 one?

Expanded on this in lines 237-243 of the Results: “In Q1, all three stimuli were presented an equal number of times (30 presentations/stimulus, P(s) = 0.33 for all stimuli), while in Q2, one randomly chosen stimulus appeared three times more frequently than the others (90 presentations or P(s) = 0.6 for one stimulus, 30

presentations or P(s) = 0.2 for the other two). Therefore, for this task only, there are overall fewer trials in Q1 (90 trials) than in Q2 (150 trials). By ensuring that the low-frequency stimuli in Q2 were presented an equal number of times in both conditions, we enable a direct comparison of choice biases associated with those stimuli across conditions."

…and in each figure caption of Supporting Fig S2-S4: "...Policy complexity, average reward, and response time (RT) as a function of trials. Response time as a function of policy complexity. Note that policy complexity, average reward, and RT are computed via a sliding window of 30 trials. The running average in each plot is therefore truncated to 30 trials fewer than the total number of trials as there are not enough elements to fill the window at endpoints."

Note that we had originally written that there were 20 presentations/stimulus for most tasks and conditions (besides the one state in Task 1) but it was actually 30 / stimulus, which is now corrected.

Reviewer #3

This paper presents a behavioral experiment testing people's tendency to favor simplified action-selection policies. The experiment includes (at any given time) 3 possible visual cues (states) and 3 available actions with varying probabilities of delivering a binary reward outcome. The general finding is that people show a bias toward actions with a high overall reward probability rather than solely relying on the action's state-specific reward probability. The paper recaps an appealing and well-developed theoretical framework for quantifying and modulating policy complexity. The specific empirical findings are that (1) an action that is rewarded in a frequently occurring state tends to be erroneously selected in other states; (2) if two actions are rewarded in a given state, people tend to favor the one that is also rewarded in other states; and (3) time pressure increases a bias toward picking actions that are rewarded in other states.

Overall, I think the paper does a nice job of outlining the policy compression model and presenting prospective empirical evidence that supports the model. Some comments:

1. I would suggest mentioning basic characteristics of the participant sample early in the results section (n=200, run online). The methods section should report demographics of the sample and the duration of the experiment.

We elaborated on line 188 in the Results section: "Using Amazon Mechanical Turk, we collected data from 200 subjects who participated in our online behavioral experiment."

…and on lines 821-825 in the Methods section: "In accordance with a power analysis run on a pilot sample, we collected data from two-hundred (N=200; 136 male) subjects who completed our study on Amazon Mechanical Turk and received monetary compensation. Subjects were paid a base pay of $4 and a performance bonus of up to $4 for completing the task. Subjects took, on average, 35 minutes to complete the entire experiment, and their average payout was $6.66. No subjects were excluded."

Gender was the only demographic we collected.

2. I wonder if it would be possible to include further discussion in the discussion section about other potential mechanisms for perseveration-like effects. It's useful to see that the simple RL and RL-WM models aren't capable of producing such effects, but what are some other ways a bias toward repeating frequent/recent previous actions might originate? An example might be a motor habit or sequential effect. In task 2, sticking with the single always-correct action could be a robust strategy against errors in state identification or

state-specific credit assignment. A motivation in terms of avoiding errors seems possibly distinct from a direct or intrinsic complexity cost.

A motivation such as avoiding errors could simply be recast as the inverse of reward, which would then make the optimization problem the same as maximizing reward (minimizing errors) under some capacity constraint. While we agree that some other forms of perseveration may co-occur with those that arise from capacity limits, their origins are beyond the scope of this paper.

We now expand upon this point in lines 514-521 the Discussion: “Finally, we note that a majority of subjects' behavior deviated from the optimal reward-complexity trade-off curve, which indicates additional sources of error and bias that are not due to the marginal action probability. For example, it is certainly possible that other forms of perseveration (e.g., repeating actions that do not have high marginal probability, such as in sequential effects or motor habits) may co-occur with those that arise from capacity limits, but their origins are beyond the scope of this paper. Future experiments could be uniquely designed to tease apart different sources of perseveration-like effects.”

3. The paper occasionally refers to "individuals with low complexity" (line 557 and elsewhere). Although policy complexity is shown to respond to contextual factors (such as time pressure), this phrasing seems to suggest it may also be partially trait-like. This could be worth some explicit discussion. It also might be possible to show some preliminary evidence on this question by testing for a correlation between tasks 1 and 2 in policy complexity or other indices of policy compression.

Great point! We now address this in line 439-452 with an additional figure (Fig 9): “Finally, we asked whether policy complexity reflected an individual's fixed resource that remained roughly constant across tasks, or whether it varied across tasks. On average, subjects' policy complexity in the control condition (Q1) remained roughly constant across tasks (Fig 9A) [one-way ANOVA: $F(2,597) = 1.032$, $p = 0.357$]. Additionally, we found a moderate positive correlation between individual policy complexities across tasks (Fig 9B) [Pearson's correlation coefficients ranging from 0.432 to 0.573, all $p<0.001$], which shows that subjects used roughly the same amount of cognitive resources for each task. Subjects' empirical policy complexity may reflect an individual's actual cognitive capacity limit (in a trait-like fashion), or simply reflect the amount of cognitive effort that the individual has chosen to allocate for the experiment tasks. While our experiment was not designed to tease apart these two possibilities, future work may consider investigating how independent, trait-like measures such as working memory capacity could predict individuals' policy complexity in experimental tasks. In either case, we know from our results that policy complexity is flexible to some degree within an individual, as it can be shaped by the task structure itself and by exogenous factors such as time pressure and memory load.”

4. Within each task, correlations are reported between policy complexity and action bias. I'm unsure about the independence of these two variables if they are both estimated from the same behavioral data. It seems that an increase in the action bias would naturally tend to reduce policy complexity (although the reverse may not be true, assuming policy complexity could be reduced in arbitrary ways that wouldn't map onto the action bias).

We now address this in lines 411-417: “While action bias and policy complexity are estimated from the same behavioral data, their relationship is not entirely tautological: though it is unlikely for a high complexity policy to produce high action bias, it is possible to have a low complexity policy (e.g., taking random actions regardless of state) without being biased towards the action with highest marginal probability. Our analysis here shows the relationship between action bias and complexity becomes more pronounced by directly manipulating the action distribution (as in Task 2), rather than the state distribution (as in Task 1).”

5. I was a little unclear on the specific basis for favoring the "capacity-value" model over the "capacity" and "value" models (line 321). I see that some of the plots of that model's dynamics in the supplemental figures look more similar to the data than the other models do, but would it be possible to say more about the specific qualitative patterns that drive the preference for that model?

We've now added more details in Supporting Figure 3 (Task 2) caption: "In the data, the policy complexity is similar across conditions and increases slightly over the course of the task. While the Capacity-Value model (B) captures this between-condition similarity, the Value (C) and Capacity (D) models predict diverging policy complexities."

We've also added to the Supporting Figure 4 (Task 3) caption: "In the data, policy complexity remains roughly constant in both conditions, though it is overall higher in Q1 than in Q2. This trend is successfully mirrored by the Capacity-Value model (B). In the Value (C) model, the complexity difference between conditions is much smaller, while in the Capacity (D) model, complexity starts at the same point for both conditions and diverges with learning. Additionally, average reward steadily increases for both conditions, though it is always higher on average in Q1 than Q2. This overall difference in reward, although less pronounced, is captured by the Capacity-Value model but not by the Capacity and Value only models."

6. In the exposition of the model, policy complexity is defined as an expectation of policy cost over states and actions. I would benefit from a little further clarification of the "policy cost" term (line 148); does it index the cost for a specific pairing of state and action, such that the policy cost in a given state differs depending on which action is taken? In terms of the effect of policy cost on RT (line 190), does this imply the RT will be faster if the agent selects an action with a lower state-specific probability (relative to its marginal probability)?

Yes, exactly!! Thank you for providing that simple explanation, we have added the following clarification in lines 145-149:

"First, we assumed that RT is monotonically related to the policy cost, which indexes the cost of taking a specific action a in the current state s by quantifying the deviation of the state-specific action policy $\pi(a|s)$ from the marginal action probability $P(a)$: $\log \pi\theta(a|s)/P(a)$. This implies that the RT will be slower in trials where the agent selects an action with a higher state-specific probability relative to its marginal probability."

and in lines 632-635: "The policy cost indexes the cost of taking a specific action a in the state s by quantifying the deviation of the current, state-specific action policy pi(a|s) from the marginal action probability P(a). In this way, the policy cost captures the amount of state-specific information used to select a particular action in a single trial or time step."

Minor: in the Figure 2 caption, "right" and "left" labels seem to be switched.

Thanks for catching! Fixed.

One note to Reviewer 3: You had marked "No" to the question of whether data and code was made fully available. I have made the data available under the "Data Availability Statement" part of the PLOS submission system. But I also linked to the code and data in the manuscript itself in lines 846-848, under the "Methods" section.
